# The study on health of older people in Germany (Health 65+): Design and first results

**DOI:** 10.1093/eurpub/ckac131.563

**Published:** 2022-10-25

**Authors:** J Fuchs, B Gaertner, H Perlitz, T Kuttig, C Scheidt-Nave, Study Group Health 65+

**Affiliations:** Department of Epidemiology and Health Monitor, Robert Koch Institute, Berlin, Germany

## Abstract

**Background:**

Integrating older persons into continuous national public health monitoring is crucial but challenging. Health 65+ is the first national health interview and examination survey in Germany specifically tailored to the needs of the population 65+ years.

**Methods:**

Health 65+ is based on two-stage stratified random sampling from 128 local population registries. It is based on a previously tested three-step procedure to contact the study population (letter, telephone, home visits). 12,448 individuals 65+ years were invited for survey participation between June 2021 and April 2022. Participation comprised answering a baseline-questionnaire/interview covering a consented set of key health indicators including SARS-COV2 infections, and 3 follow-ups. After one year participants are invited to an examination (e.g. blood pressure, grip strength, cognitive function) during a home visit. All-cause mortality, health insurance data and information on social and built environment will be linked to survey data. The preliminary data set comprises 3,107 baseline participants.

**Preliminary results:**

Only few individuals were excluded for survey participation as they had deceased before invitation, moved to an unknown residence or had insufficient German language skills. Of the adjusted gross sample, 32% took part in the survey (47.9% women, mean age 78.8 years). Only 3.3% of the participants did not receive any vaccination against COVID-19, with no differences between gender or age-groups. 3.5% had already experienced a laboratory-confirmed SARS-CoV-2 infection.

**Conclusions:**

Health 65+ collects information that cannot be obtained from any other data sources. In combination with information from routine health data and official health statistics, the results will aid health policy planning and implementation research to improve health and wellbeing of older people in Germany. For example, preliminary results show, that vaccination acceptance was high in persons 65+ years in Germany.

**Key messages:**

• Health 65+ will provide health data of people 65+ years in Germany.

• The results will aid health policy planning.

